# A Study of the Structure of Japanese University Students’ Awareness of Long-Term Care Socialization

**DOI:** 10.3390/healthcare9091106

**Published:** 2021-08-26

**Authors:** Xuxin Peng, Hisae Nakatani, Masayuki Kakehashi, Masatoshi Matsumoto

**Affiliations:** 1 Department of Community and Public Health Nursing, Graduate School of Biomedical and Health Sciences (Health Sciences), Hiroshima University, Hiroshima 7348553, Japan; hinakata@hiroshima-u.ac.jp; 2 Department of Health Informatics, Graduate School of Biomedical and Health Sciences (Health Sciences), Hiroshima University, Hiroshima 7348553, Japan; kakehashi@hiroshima-u.ac.jp; 3 Department of Community—Based Medical System, Graduate School of Biomedical and Health Sciences (Medicine), Hiroshima University, Hiroshima 7348553, Japan; matmo10@jb3.so-net.ne.jp

**Keywords:** long-term care, socialization, awareness, structure, student, Japan

## Abstract

This study aimed to examine the structure of the awareness of long-term care socialization by focusing on the younger generation’s awareness in order to improve a sustainable long-term care system. A questionnaire that assessed personal attributes and awareness of long-term care socialization was administered. In total, the answers of 209 students (48.4%) were collected for factors related to the awareness of long-term care socialization extracted through exploratory factor analysis. Additionally, the responses 149 students (56.7%) were collected for the construct validity verified through confirmatory factor analysis. According to the exploratory factor analysis, awareness of long-term care socialization included 10 items and three factors: “care burden when caring for family”, “feelings about leaving family care to society”, and “sense of responsibility to care for family as a member of the family”. The goodness-of-fit model in the confirmatory factor analysis proved the awareness of long-term care socialization scale’s construct validity. The structure of the awareness of long-term care socialization included three factors: “care burden when caring for family”, “sense of responsibility to care for family as a member of the family”, and “feelings about leaving family care to society”. This study demonstrated the scale’s good reliability and validity.

## 1. Introduction

The aging population is a common issue worldwide [1,2,3]. In many developed countries, and not only in Japan, the aging population, growing healthcare costs, and rising burden of chronic diseases call for an improvement in the healthcare system [4]. As in Europe, although increasing immigration and alternative efforts to increase fertility have been suggested to deal with the aging problem, neither of these have been pursued within realistic boundaries [5]. It is not only developed countries, but also developing countries that face this severe challenge. For example, in Brazil, which has experienced an unprecedented speed of the aging of the population, it has caused many social, economic, and public health problems, and it requires measures that can minimize or even solve these problems [6]. Furthermore, the Chinese population constitutes 18% of the world’s population, and 164.5 million Chinese citizens were aged 65 and over in 2019. China has become an aging society, and as it continues to age, the burden borne by the current families and public healthcare systems will be exacerbated [7]. As Japan has the highest aging rate, it is important to focus on its aging problem. Additionally, it is necessary to reduce the burden on the family and to create a system that allows society to take care of the elderly people. Moreover, it is important to determine the awareness of younger people who will be responsible for long-term care in the decades ahead and to consider the future of long-term care.

With a declining birth rate and aging population in Japan, the percentage of elderly people over 65 years old reached 28.4% in 2020, which was the highest worldwide, and it is expected to reach 38.3% in 2055 [8].

Elderly people have a higher incidence of disease than other age groups [9]. Moreover, care for elderly people will become a significant burden on healthcare professionals even if they have specialized knowledge and experience, and the economic burden will increase [10]. Furthermore, the rise in the number of elderly people who need nursing care and the cost of their care services will endanger not only long-term care insurance, but also Japanese society itself [11].

Long-term care insurance in Japan, which was established in 2000, is based on the slogan “from family care to social care” and its target is “to maintain an independent daily routine according to each person’s unique level of dignity and ability”. It aims to support people in need of long-term care and prevent them from becoming dependent [12,13]. In addition, the long-term care insurance’s target of “long-term care socialization” has been explained by Ishii as “socializing long-term care awareness, promoting care services, and systematizing care knowledge and care technique in order to liberate family members” [14]. The “awareness of long-term care socialization” refers to the awareness of leaving care to specialists and searching for people outside of the family who can help when they need to receive or provide long-term care [14]. The community-based integrated care system, which was established in 2017, requires municipalities, as the insurers of the long-term care insurance system, as well as prefectures, to establish a system that is based on regional autonomy and independence [15].

Japan’s total fertility rate has generally been on a long-term decline, decreasing from 2.135 in 1970 to 1.42 in 2018, which was a historical low. Due to the declining fertility rates among those in their twenties, especially in those under 24 years old [16], university students attach great importance to family-centered long-term care even though long-term care insurance has been established [17]. This shows that, despite the rapid increase in the aging population, there has been no change in the awareness of younger Japanese people; on the contrary, it is decreasing [18]. Affected by the declining birth rate, aging population, and nuclear family, smaller nuclear families may face the problem of a lack of manpower for providing care for their family members, which indicates that long-term care socialization is a prominent social problem [19].

It is important to focus on its aging problem. Additionally, it is necessary to reduce the burden on the family and to create a system that allows society to take care of the elderly people. Previous studies on long-term care socialization focused heavily on the policy research and other age groups [20,21], although there is a previous study focused on university students’ awareness of long-term care socialization, but only one item was used to evaluate the awareness of long-term care socialization [22]. Therefore, it is important to clarify the structure of the awareness of long-term care socialization, which will contribute to determining the awareness of younger people who will be responsible for long-term care in the decades ahead, as well as to consider the future of long-term care. The innovation of this study is that, as the country with the highest aging population rate, Japanese young people’s awareness of long-term care socialization has been explored, which is helpful for development of long-term care socialization worldwide.

Referring to the characteristic of family care behavior [23], the definition of “care” in the current study refers to the necessary daily care involved in medical treatment, such as dealing with meals, cleaning, excretion, laundry, medication, and so on.

This study aimed to examine the structure of the awareness of long-term care socialization by focusing on the awareness of the younger generation in order to improve the sustainable long-term care system.

## 2. Materials and Methods

### 2.1. Design and Sample

In order to examine the structure of the awareness of long-term care socialization, data were collected twice from two universities in City A. First-time data collection was collected for factors related to the awareness of long-term care socialization extracted through exploratory factor analysis. Second-time data collection was collected for the construct validity verified through confirmatory factor analysis. The first-time data collection consisted of 432 students and was conducted from September to November 2020. The second-time data collection consisted of 263 students and was conducted from December 2020 to June 2021. After receiving permission from the professors of the class, we explained the research content and ethics to the students and sent a QR code for the online research before or after the class. The response surveys were collected two weeks later via the internet.

### 2.2. Measures

The questionnaire included items about the students’ personal attributes and their awareness of long-term care socialization. The items about their personal attributes focused on their gender, age, nationality, current grade, major (faculty of a health school, such as nursing, rehabilitation, medical, or faculty of a non-health school), and thoughts about the importance of social welfare policies for long-term care (1 = very unimportant, 2 = unimportant, 3 = neutral, 4 = important, 5 = very important).

In the survey, 15 items about the awareness of long-term care socialization were created based on 20 statements that described the phenomenon or thoughts about “awareness of long-term care”, “long-term care socialization”, “awareness of long-term care socialization”, and included sentences about “leaving care to specialists” and “finding people outside of the family to help when they need to receive or provide long-term care”. The grade of the awareness of long-term care socialization was classified into five categories (1 = strongly disagree, 2 = disagree, 3 = neutral, 4 = agree, 5 = strongly agree) (Supplementary Materials).

### 2.3. Analytical Strategy

The data collected from the first-time data collection were used for the item analysis. Before conducting a factor analysis, an item analysis of the awareness of long-term care socialization was performed. The average values and standard deviations of the 15 items were computed, and the ceiling and floor effects were evaluated. Next, the Spearman’s correlation coefficient was used for the item-total correlation analysis.

After the item analysis, exploratory factor analysis (maximum likelihood method, promax rotation) was used to evaluate the scale’s content structure, and Cronbach’s α coefficients were computed to estimate the scale’s internal consistency. In addition, the criterion validity was assessed by examining the Spearman’s correlation coefficients between the awareness of long-term care socialization total score, the subscale scores, and the thoughts about the importance of social welfare policies for long-term care. Items exhibiting factor loads of ≥0.3, and α ≥ 0.6 were examined as a standard for the content structure using IBM SPSS Statistics for Windows version 27 (IBM Corp., Armonk, NY, USA) [24].

The construct validity was verified and tested by confirmatory factor analysis (maximum likelihood method) using the data collected from the second-time data collection. AMOS 27 (IBM Corp., Armonk, NY, USA) was used to evaluate the model fit, goodness-of-fit index (GFI), adjusted goodness-of-fit index (AGFI), comparative fit index (CFI), and root-mean-square error of approximation (RMSEA).

## 3. Results

### 3.1. Personal Attributes

A total of 209 students’ (48.4%) responses were collected in the first-time data collection and a total of 149 students’ (56.7%) responses were collected in the second-time data collection. The personal attributes of the students are listed in Table 1. In the first-time data collection, the students comprised 56 males (26.8%) and 153 females (73.2%), with an average age of 20.06 ± 1.49 years old. As for the major of students, 120 (57.4%) were in a health school faculty, while 89 (42.6%) were in a non-health school faculty. Regarding the current grade, 71 students (34.0%) were in first grade, 44 students (21.1%) were in second grade, 82 students (39.2%) were in third grade, and 12 students (5.7%) were in fourth grade. As for the thoughts about the importance of social welfare for long-term care, the average score was 1.58 ± 0.811. In the second-time data collection, the students comprised 34 males (22.8%) and 115 females (77.2%), with an average age of 20.03 ± 2.45 years old. As for the major of students, 112 (75.2%) were in a health school faculty, while 37 (24.8%) were in a non-health school faculty. Regarding the current grade, 63 students (42.3%) were in first grade, 20 students (13.4%) were in second grade, 51 students (34.2%) were in third grade, and 15 students (10.1%) were in fourth grade. As for the thoughts about the importance of social welfare for long-term care, the average score was 1.54 ± 0.722.

### 3.2. Exploratory Factor Analysis

The average score of the items about long-term care socialization ranged from 1.58 to 4.57 and all the standard deviations of the items were within 1. However, item 4 (M = 4.57, SD = 0.751) was removed due to a ceiling effect. In order to ensure that higher values were indicative of a higher level of awareness of long-term care socialization, the scores of eight items were reversed according to the reverse correlation between the items. The item–total correlation analysis had a range of 0.139 to 0.620. Item 11 (*r* = 0.139, *p* = 0.045) and item 15 (*r* = 0.175, *p* = 0.011) were removed because of the lower item–total correlation after carefully considering whether these items were able to reflect the content of the awareness of long-term care socialization.

The exploratory factor analysis of the 12 items (maximum likelihood method, promax rotation) showed that they had factor loadings of >0.3, except for two items. After removing these two items, the factor analysis was conducted again. Ten items were extracted that were included in three factors (Table 2), and the cumulative rate of 59.316% was found before the promax rotation.

The first factor contained three items that captured the construct about the feelings about the physical, mental, and economic aspects of the long-term care burden when caring for family. Therefore, the first factor was termed “care burden when caring for family”. The second factor comprised four items that represented the construct about the sense of responsibility to care for family as a member of the family, such as “I am my parent”, “it is natural”, “returning the favor”, and “not choosing to leave family care to others”. Therefore, the third factor was termed the “sense of responsibility to care for family as a member of the family”. The third factor contained three items that reflected the construct about the feelings about leaving family care to professionals and social support, such as being worried about being evaluated by others, the burden on society, and not wanting to leave family care to others. Furthermore, the items of the second factor were reversed items. Therefore, the second factor was termed “feelings about leaving family care to society”.

The total scores of the awareness of long-term care socialization and the three subscales were also calculated. The “awareness of long-term care socialization” score was 24.354 ± 5.244, the “care burden when caring for family” score was 7.751 ± 22.411, the “sense of responsibility to care for family as a member of the family” score was 10.641 ± 2.822, and the “feelings about leaving family care to society” score was 5.962 ± 1.997. In addition, the Cronbach’s α coefficient of the 10 items was 0.774, with “care burden when caring for family” being 0.845, “sense of responsibility to care for family as a member of the family” being 0.729, and “feelings about leaving family care to society” being 0.674 (Table 3).

### 3.3. Criterion Validity

The correlation indicated that there was a significant positive relationship between the awareness of long-term care socialization and thoughts about the importance of social welfare policies for long-term care, while all the subscales were also significantly and positively related to thoughts about the importance of social welfare policies for long-term care (Table 4).

### 3.4. Confirmatory Factor Analysis for Construct Validity

**Figure 1:**
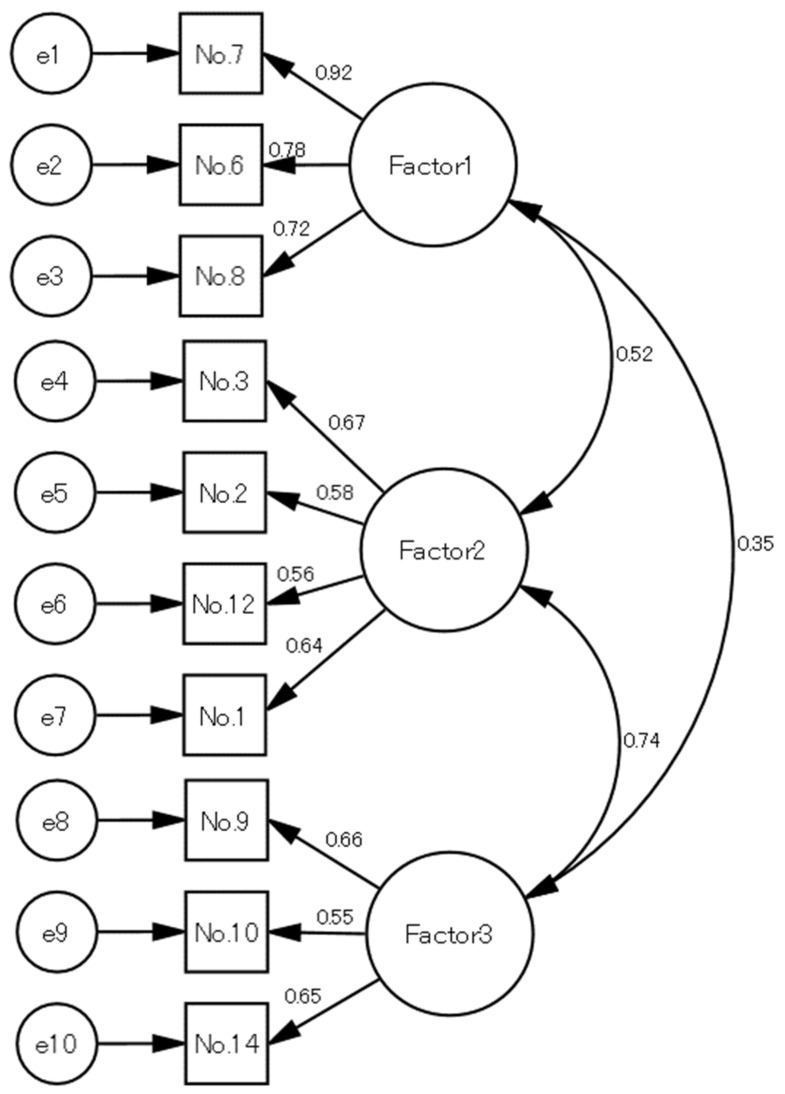
Confirmatory factor analysis of the awareness of long-term care socialization. Numeric values were standardized estimates: goodness-of-fit: χ^2^/*df* ratio = 1.973, goodness-of-fit index = 0.908, adjusted goodness-of-fit index = 0.871, comparative fit index = 0.918, and root-mean-square error of approximation = 0.081. No. = the number of each item, e = error correlation for each item, Factor 1 = care burden when caring for family, Factor 2 = sense of responsibility to care for family as a member of the family, and Factor 3 = feelings about leaving family care to society. The circles represent the latent variables, and the boxes represent each observed variable. Values in the middle of the two arrowhead lines represent the correlation between the factors. Values in the arrows that point from the factors to the observed values represent the loadings of each of the observed values in the corresponding factor. The values above each observed variable represent the variance explained by the factor.

In this study, confirmatory factor analysis was used as part of the structural equation modeling, and the model for describing the structure of the awareness of long-term care socialization was built on three factors. The results of the confirmatory factor analysis were as follows: chi-squared/degrees of freedom ratio (χ^2^/*df* ratio) = 76.928/39 = 1.973, GFI = 0.908, AGFI = 0.871, CFI = 0.918, and RMSEA = 0.081. AGFI was slightly low and RMSEA was slightly high, and the factors associated with long-term care socialization were clarified. (Figure 1).

## 4. Discussion

The structure of the awareness of long-term care socialization is comprised of three factors: the “care burden when caring for family”, “feelings about leaving family care to society”, and “sense of responsibility to care for family as a member of the family”. We will consider each of these factors in turn.

Extraction of the factors of “care burden when caring for family” confirmed that care burden could promote the awareness of long-term care socialization. Furthermore, the extraction of the factors of “feelings about leaving family care to society” and “sense of responsibility to care for family as a member of the family” showed that the awareness of long-term care socialization was negatively influenced by students’ feeling and their responsibility to care for family considering these two factors comprised by reverse items.

The burden of long-term care has been found to be an important factor influencing families’ decisions about whether to continue providing home care or turn to a long-term care facility [25]. A family caregiver might be overwhelmed and the dynamics of caring will be strongly influenced due to the care burden [26]. Care burden can have devastating effects on caregivers and expose them to various diseases. The burden experienced by caregivers can lead to serious diseases [27]. Thus, reducing the burden of long-term care is a meaningful issue related to the quality of life of family caregivers. A previous study reported that usage of long-term care insurance care services successfully relieves the burden on family caregiver [28]. Especially for young people, since they require more time for their own business, long-term care socialization is urgently needed. However, many elderly people in Japan still want to be cared for by their own child [29]; even though long-term care insurance was developed in 2000, the culture that emphasizes filial piety can lead to a more severe care burden when family caregivers need to provide care frequently [30]. According to the Annual Report on the Ageing Society 2021, only 12.1% of families selected a long-term care facility as the main caregiver [31]. Thus, we are convinced that the awareness of long-term care socialization needs to be improved. With the improvement of awareness of long-term care socialization, more and more families might select a long-term care facility, which can improve the promotion of social care and be beneficial to promote the institutional and community-based long-term care services, which is under the slogan “from family care to social care”, which would additionally reduce the care burden of the family caregiver.

Furthermore, the sense of responsibility to care for the family and feelings about leaving family care to society would decrease the awareness and tendency of long-term care socialization. In Japan, as in many Asian countries, there is a strong traditional filial piety obligation that is rooted in the coexistence of generations [32,33]; the students held the feeling that, as a member of family, caring for their parents is “natural” and “repayment”. There is a resistance to leaving family care to others rather than to family members influenced by Confucianism [34], traditional values strongly emphasize filial piety and righteousness in which family relations are structured hierarchically based on age and generation [35], young people are supposed to be responsible for the family care, and if not, they may feel shame or loss of face [36]. Thus, we believe that, with the development of the healthcare system and society, traditional family relationships and the awareness of long-term care socialization need to be improved. As such, family-based care support might transfer to community-based care support, which contributes to a long-term care policy. Furthermore, improvement of the awareness of long-term care socialization might be conducive to a long-term care policy, contributing to the increasing societal roles in elderly care [37].

Moreover, the results showed that thoughts about the importance of social welfare policies for long-term care were significantly and positively correlated to the awareness of the long-term care socialization scale and subscales, which indicated that the more people considered the importance of social welfare policies for long-term care, the greater the increase in the awareness of long-term care socialization, which was likely due to the fact that people who had a high level of awareness of long-term care socialization noticed that social welfare policies played an important role in long-term care socialization, and it enabled them to be certain of the importance of social welfare policies for long-term care. However, the scores of “thoughts about the importance of social welfare for long-term care” were 1.58 ± 0.811 and 1.54 ± 0.722, which showed that students did not think social welfare was important to long-term care. In this study, it is showed that “sense of responsibility to care for family as a member of the family” was related to the awareness of long-term care socialization, they tended to care for their family by themselves rather than specialists or people outside. Hence, we believe that the promotion of education about social welfare is needed.

The Cronbach’s alpha value of the awareness of long-term care socialization was 0.774, which exceeded the 0.70 level; this indicated the high internal consistency reliability of this newly developed professionalism assessment scale [38]. In addition, the Cronbach’s alpha values of the three sub-dimensions were 0.845 for “care burden when caring for family”, 0.729 for “sense of responsibility to care for family as a member of the family”, and 0.674 for “feelings about leaving family care to society”. Thus, this indicated that the scale of awareness of long-term care socialization had good reliability.

Regarding the results of the awareness of long-term care socialization model’s goodness-of-fit, the values were of 0.908 for the GFI, 0.871 for the AGFI, and 0.918 for the CFI. Although AGFI was slightly lower, GFI and CFI were over 0.9, and the value of 0.081 for the RMSEA was slightly higher than 0.08 but below 0.1, which was acceptable. Therefore, overall, a good model fit was obtained, and the scale of the awareness of long-term care socialization had overall construct validity.

In the future, the structure of awareness of long-term care socialization could be further clarified through a quantitative study based on the funding in this study. If the awareness of long-term care socialization is demonstrated, it is quite helpful for the reform of long-term care insurance and is beneficial for solving the aging problem. To be utilized as a scale, we need to enlarge the item pool and modify the expressions of the questions to increase the reliability and validity of the scale. Furthermore, only Japanese university students were consulted in this study, so other age groups or people in other countries need to be take into consideration in the continuous research.

## 5. Conclusions

The three factors associated with the awareness of long-term care socialization were “care burden when caring for family”, “sense of responsibility to care for family as a member of the family”, and “feelings about leaving family care to society”. This study assessed the awareness of long-term care socialization scale, which showed good reliability and validity.

## Figures and Tables

**Table 1:** Personal attributes of the students.

Items	First Time (N = 209)			Second Time (N = 149)		
	N (%)	Mean	SD	N (%)	Mean	SD
Age (years)		20.06	±1.492		20.03	±2.455
Thoughts about the importance of social welfare for long-term care		1.58	±0.811		1.54	±0.722
Gender						
Male	56 (26.8)			34 (22.8)		
Female	153 (73.2)			115 (77.2)		
Major						
Faculty of a health school	120 (57.4)			112 (75.2)		
Faculty of a non-health school	89 (42.6)			37 (24.8)		
Current Grade						
First Grade	71 (34.0)			63 (42.3)		
Second Grade	44 (21.1)			20 (13.4)		
Third Grade	82 (39.2)			51 (34.2)		
Fourth Grade	12 (5.7)			15 (10.1)		

**Table 2:** Exploratory factor analysis of the awareness of long-term care socialization.

Question Items	Factor 1	Factor 2	Factor 3
Factor 1: Care burden when caring for family			
No. 7: Parental care is a physical burden on you	**0.872**	−0.075	0.119
No. 6: Parental care is a mental burden on you	**0.835**	0.051	−0.032
No. 8: Parental care is an economic burden on you	**0.709**	0.058	−0.112
Factor 2: Sense of responsibility to care for family as a member of the family			
No. 3: I cannot leave my parents’ care to others because they are my parents	−0.049	**0.869**	−0.027
No. 2: It is natural that my parents are cared for only by family members	0.088	**0.585**	−0.084
No. 12: I do not want to leave the care to anyone other than my family	−0.018	**0.417**	0.255
No. 1: Caring for my parents is a repayment for raising me	0.045	**0.391**	0.280
Factor 3: Feelings about leaving family care to society			
No. 9: Leaving my parents’ care to others puts a burden on society	−0.050	−0.088	**0.739**
No. 10: I am worried about others’ evaluation of me when I leave my parents’ care to them	0.045	0.021	**0.726**
No. 14: I will not seek help from others until I reach the limit of my nursing ability	−0.044	0.261	**0.381**

The maximum likelihood method (Promax rotation) was used. Numeric values were factor load of each item.

**Table 3:** Reliability of the awareness of long-term care socialization.

Components	Mean	SD	Cronbach’s α
Total score of the scale	24.354	5.244	0.774
Factor 1: Care burden when caring for family	7.751	2.411	0.845
Factor 2: Sense of responsibility to care for family as a member of the family	10.641	2.822	0.729
Factor 3: Feelings about leaving family care to society	5.962	1.997	0.674

**Table 4:** Criterion validity of the awareness of long-term care socialization.

Components	Thoughts about the Importance of Social Welfare Policies for Long-Term Care	
	γ	*p*
Total score of the scale	0.296 **	0.000
Factor 1: Care burden when caring for family	0.280 **	0.000
Factor 2: Sense of responsibility to care for family as a member of the family	0.137 *	0.047
Factor 3: Feelings about leaving family care to society	0.245 **	0.000

γ = Spearman’s correlation coefficient; *p = p*-value; * *p* < 0.05, ** *p* < 0.01.

## Supplementary Materials

The following are available online at https://www.mdpi.com/article/10.3390/healthcare9091106/s1, Figure S1: Process of questionnaire construction.
