# Supplementary material for: A Study of the Structure of Japanese University Students’ Awareness of Long-Term Care Socialization
Source: Healthcare (Basel). 2021 Aug 26;9(9):1106. doi: 10.3390/healthcare9091106 (PMC8467874; doi:10.3390/healthcare9091106)
Supplement: Supplementary file 1 [file healthcare-09-01106-s001.zip › healthcare-1337760-SI.pdf]

Process of questionnaire construction

A scale was developed according to the following process (Figure S1).

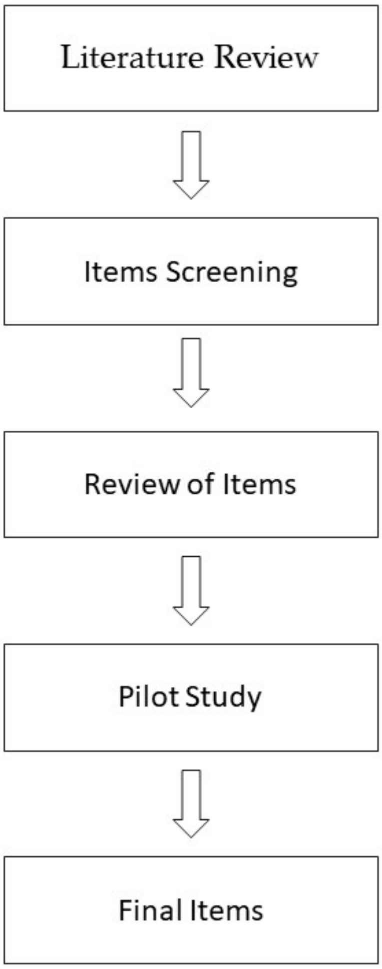

Figure S1. Process of questionnaire construction.

Literature Review

A literature review was conducted on the database in Japanese, English and Chinese. The literature review was mainly focused on the Japanese articles using the search terms “awareness”, “long-term care”, “elderly care”, “socialization” and “social care”. After removing the unrelated research, 20 articles were used to build an item pool.

Item Screening

After reading the articles carefully, the contents related to “long-term care socialization” and “awareness of long-term care socialization” or “leaving care to specialists” and searching for people outside of the family” were extracted. Then, similar sentences were merged and 20 items were selected.

Review of items

The constructed items were reviewed twice by six experts with an academic background in

long-term care insurance, statistics or healthcare. The experts evaluated whether items were valid and items' clarity, conciseness, and redundancy. For the result, 2 items were removed and 6 items were merged into 3 items. In addition, this produced a total of 15 items.

Pilot Study

A pilot study was conducted to evaluate the degrees of understanding concerning the sentences and vocabularies. The data for the pilot study were gathered from 10 Japanese university students, the time required to complete the questionnaire was also surveyed.

Final Item

Therefore, 15 items about the awareness of long-term care socialization were created and the grade of the awareness of long-term care socialization was classified into five categories (1 = strongly disagree, 2 = disagree, 3 = neutral, 4 = agree, 5 = strongly agree).
